# Poly (Ethylene Glycol) Methyl Ether Methacrylate-Based Hydrogel and Cerium(IV) Oxide Nanoparticles as Ophthalmic Lens Material

**DOI:** 10.3390/mi12091111

**Published:** 2021-09-16

**Authors:** Min-Jae Lee, Seon-Young Park, A-Young Sung

**Affiliations:** 1 Department of Optometry, Jeju Tourism University, Jeju 63063, Korea; tomssamac@naver.com; 2 Department of Optometry & Vision Science, Daegu Catholic University, Gyeongsan 38430, Korea; ssun_419@naver.com

**Keywords:** cerium(IV) oxide nanoparticles, hydrogel, ophthalmic lens material, UV-light blocking, tensile strength

## Abstract

The functional hydrogel lens containing 2-hydroxyethylmethacrylate (HEMA) was manufactured by thermal polymerization. The physical properties of the produced hydrogel lens were measured and analyzed. In this study, HEMA, ethylene glycol dimethacrylate (EGDMA), and azobisisobutyronitrile (AIBN) were used for thermal copolymerization. Additionally, poly (ethylene glycol) methyl ether methacrylate (PEGMEMA), 3-(Triethoxysilyl) propyl isocyanate (TEPI), and cerium(Ⅳ) oxide nanoparticles were used as additives to make a functional hydrogel lens. The mixture was heated at 100 °C for 90 min to produce the hydrogel ophthalmic lens by the cast mold method. The resulting physical properties showed that the water content and refractive index of the sample were in the ranges of 38.06~42.11% and 1.426~1.436, respectively. The addition of cerium oxide nanoparticles lowered the contact angle and allowed the hydrogel lens to block UV light. The tensile strength was also improved by 52.13% through cerium oxide nanoparticles, and up to 123.4% by using TEPI. Based on the results of this study, the produced ophthalmic lens is suitable for durable, UV-blocking high-performance lenses.

## 1. Introduction

There has been a substantial development in the physical properties of ophthalmic lenses, as various polymers have begun to be used as materials in hydrogel lenses [1,2,3,4]. After Otto Wichterle and Drahoslav of the Czechosloviakia National Polymer Institute developed a hydrophilic polymer, polyhydroxyethyl methacrylate (PHEMA), in 1955, contact lenses made of hydrophilic hydrogel became popular due to their favorable water content and comfort. As various polymers began to be used as materials for hydrogel lenses, such lenses have become a common vision-correcting tool. Various studies are ongoing on the further application of polymers as hydrophilic lens materials [5]. Despite such studies, some limitations have been found in the material properties. Therefore, many studies are currently being conducted based on 2-hydroxyethyl methacrylate (HEMA), a fundamental monomer of a hydrophilic lens material with a hydroxyl group. Generally, for hydrogel lenses, the refractive index is lower, and the durability decreases as the water content is increased [6]. Additionally, the durability tends to become lower when the thickness of the lens is reduced to achieve high oxygen permeability. The wettability of the hydrogel lens can be assessed from the contact angle between water and the lens surface, and the durability of the contact lens can be assessed through its tensile strength. As the tensile strength and the water content of the hydrogel lens are inversely proportional, the tensile strength and wettability are significant physical properties of contact lenses [7,8]. Attention to the antibacterial activity of contact lenses is also increasing, because direct contact of hydrogel lenses with the eye may cause bacterial infection, as the human eye is one of the organs that are most vulnerable to germs. *Escherichiacoli*, *fungus*, *Bacillus pyocyaneus*, and *Staphylococcus aureus* are common microorganisms that cause ocular diseases, including keratohelcosis and conjunctivitis [9,10,11]. Additionally, the human eye is prone to ultraviolet (UV) rays [12,13], and not protecting it from UV can cause many ocular diseases, including cataract, keratitis, and conjunctivitis, and can cause loss of visual acuity [14,15,16]. The use of hydrogel lenses with a high UV-blocking rate can protect the eyes from UV, as they are expected to have better eye UV-blocking efficacy than colored lenses [17]. With UV-blocking contact lenses gaining more attention, studies on materials for lenses with a UV-blocking function are ongoing. The UV-B zone of 280–315 nm especially accelerates skin inflammation and aging, and also causes thinning of the cornea epithelium, cornea clouding, and cornea inflammation [18,19]. In fact, extreme caution is needed, as such rays can severely damage the cornea by destroying its epithelium and basal cells, and inducing the exfoliation of its epithelium cells. As compared to other lens materials, hydrogel does not significantly change the physical properties of hydrophilic lenses and has excellent UV-blocking activity, and its compatibility with other monomers must be investigated. Metal oxide nanomaterials play an important role in a wide range of biomedical applications, such as diagnostics, drug delivery, medical implants, magnetic resonance imaging, tissue engineering, and cancer treatment [20]. Today, cerium oxide nanoparticles are considered one of the most promising metal oxide nanobiomaterials [21]. Antioxidative contact lens research using cerium nanoparticles is in progress [22,23], and we tried to find their properties using different combinations of additives. Additionally, UV protection experiments using cerium nanoparticles were conducted in various fields [24,25,26,27]. In this experiment, the characteristics of cerium nanoparticles were applied to contact lenses, and the performance of lenses was improved.

## 2. Materials and Methods

### 2.1. Reagents and Materials

This study used 2-hydroxyethyl methacrylate (HEMA) as the main material of hydrogel lenses; azobisisobutyronitrile (AIBN, Junsei, Tokyo, Japan), as the initiator; ethylene glycol dimethacrylate (EGDMA), as the cross-linking agent; and poly (ethylene glycol) methyl ether methacrylate (PEGMA), 3-(triethoxysilyl) propyl isocyanate (TEPI), and cerium(IV) oxide nanoparticles (Sigma-Aldrich, Saint Louis, MO, USA), as additives for functional hydrogel lenses. A nanopowder, <50 nm particle size, was used, with 99.95% trace rare earth metal basis. Its properties include a high refractive index and dielectric constant. CeO_2_ nanopowder can be used for a variety of applications, such as biomedicine, semiconductors, and coatings.

### 2.2. Polymerization

This study used HEMA, a known main material of hydrogel lenses, AIBN as initiator, and EGDMA as a cross-linking agent, to which 1~10% PEGMA, 1~5% TEPI, and 0.1% cerium(IV) oxide nanoparticles were added. HEMA, EGDMA, AIBN, and the additives were added following the formulation ratio, sonicated for approximately 30 min to disperse the nanoparticles, and mixed for 3 h using a mixer (Vortex GENIE 2, Scientific Industries, Bohemia, NY, USA). The mixture was polymerized for 1 h under 100 °C using the cast mold method. The hydrogel sample was hydrated for 24 h in a sterilized normal saline solution, after which its physical and optical properties were evaluated, including its refractive index, optical transmittance, and water content. The experimental group that did not contain additives was named “ref”, and the group that contained cerium(IV) oxide nanoparticles was named “Ce”. The subgroups were named CeP1, CeP3, CeP5, and CeP10 according to the ratio of their added PEGMA, and the CeP10 group with optimal physical properties was further classified into CeP10_ISO1, CeP10_ISO3, and CeP10_ISO5 according to the ratio of their added TEPI. The polymerization method and the mix proportion of each hydrogel sample used in this experiment are summarized in Table 1.

### 2.3. Analysis

The ISO 18369-4:2006 standard was used as the measurement standard for the refractive index. The refractive index of the sample that was hydrated for 24 h in normal saline was measured with an ABBE Refractometer (ATAGO DR-A1, Tokyo, Japan). To measure the water content, the ISO 18369-4:2006 weighing method was used. Before the sample was hydrated, the dry lens was measured; after 24 h of hydration in water, only the moisture on the lens surface was removed. The hydrated lenses were weighed five times, and the average result was presented. For the spectral transmittance, a spectral transmittance meter (Cary 60 UV-vis., Agilent, Santa Clara, CA, USA) was used, and the transmittance values for the UV-B (280–315 nm), UV-A (315–380 nm), and visible-light (380–780 nm) regions were measured five times, respectively, and then averaged. For the wettability, the contact angle was measured using a contact angle instrument (DSA30, Kruss GmbH, Hamburg, Germany) following the Sessile drop method. For the strength of the lenses, their tensile strength was measured using the Universal Testing Machine (AGS-X, Shimadzu, Kioto, Japan), and a scanning electron microscope (FESEM; JSM-7500F+EDS, Tokyo, Japan) and an atomic force microscope (XE-100, Park Systems, Suwon, Korea) were used to observe the surface and roughness. To identify the extractables, the absorbance, pH change, and presence of potassium permanganate-reducing substances were observed. For the absorbance, a spectral transmittance meter was used to measure the absorbance of the highest wavelength for 5 runs, after which the average was presented. For the pH change, a less than 1.5 difference from the control group was determined as having no effect. For the potassium permanganate-reducing substance, a less than 2 mL difference from the control group was categorized as showing no extractables. For the antibiotic property, the antibacterial activity against *Staphylococcus aureus* and *Escherichia coli* (*E. coli*) was observed. For the experiment, 3 M Petrifilm^TM^ was used as the dry film medium, and the bacteria were added along with the lens to the normal 0.9% sodium chloride saline and hydrated for 24 h, after which the moisture on the surface was removed. The lens was put into the saline solution that was 9 times its weight and then shaken, and 1 mL of this saline solution was smeared onto a dry film and incubated for 24 h under 36 ± 1 °C. The shaking incubator (DS-210SL, Daewon Science, Bucheon, Korea) was used to incubate the bacteria.

## 3. Results and Discussion

### 3.1. Physical Properties

#### 3.1.1. Refractive Index and Water Content

**Figure 1:**
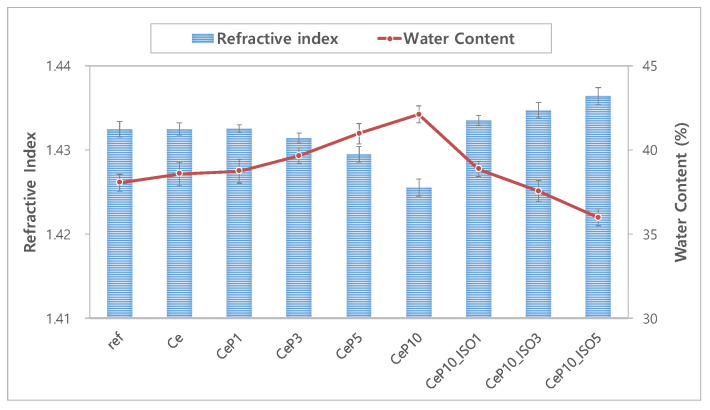
Change in refractive index and water content of samples.

The refractive index is a critical property that represents the optical characteristic of a material, and affects the refractive power of the hydrogel lens. The use of a hydrogel material with a high refractive index may reduce the thickness of the lens [28]. It is also closely associated with comfort as it affects strength. The refractive index and water content of each sample are presented in Figure 1. The refractive index of both the conventional ref and Ce samples was 1.432 and decreased to 1.426 with the addition of PEGMA. The samples with 1~5% TEPI showed a refractive index of 1.433~1.436, which is 0.01 higher than in CeP10. Generally, the sample with a high water content showed a low refractive index [29,30]. The CeP10 sample with the highest water content among the samples used in this study showed the lowest refractive index, and the CeP10_ISO5 sample, the water content of which decreased with the addition of TEPI, showed the highest refractive index [31]. These results show that adding PEGMA contributes to a higher water content, and that TEPI is a favorable monomer that enhances the refractive index [32]. Thus, these monomers can be used to manufacture highly refractive hydrogel lenses.

#### 3.1.2. Optical Transmittance

**Figure 2:**
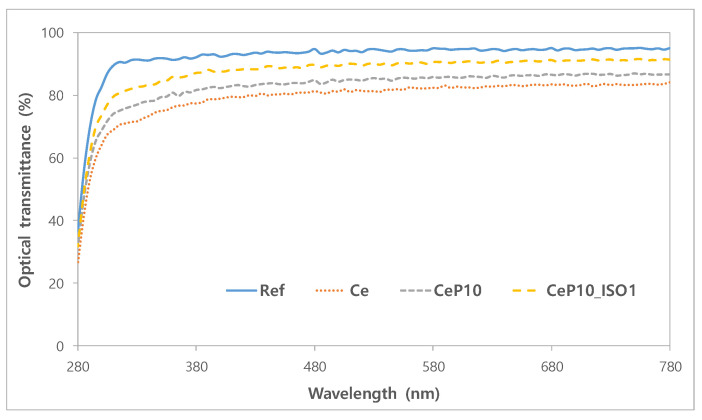
Optical transmittance of samples.

The optical transmittance of each sample was measured to determine the optical properties of the lenses. The mean visible light transmittance of ref was as high as 93.69% and was 91.04% for UV-A and 71.79% for UV-B, which shows that it does not block UV. For the mean optical transmittance of the sample that contained cerium(IV) oxide nanoparticles, the visible light transmittance was 82.50%, and 75.08% for UV-A and 57.00% for UV-B, which demonstrates the sample’s UV-blocking property. PEGMA and TEPI are seen as having the potential to improve the clarity of lenses, as they seem to improve the visible light transmittance while maintaining the UV-blocking rate. The optical transmittance rates of the formulas are summarized in Figure 2. Benzophenone substances are generally used as UV absorbents for hydrogel lenses, but their use is limited, as they change the physical properties of the lenses and affect their water content and refractive index. CeNPs, due to their high transparency in the visible light range of spectra, as well as their antioxidant, therapeutic, antibacterial and UV-shielding properties, could be a good candidate as a filling material for contact lenses [33]. As the monomer formula used in this study enhances the optical functionality without changing the physical properties of the lens, it has potential as a useful additive for contact lens materials.

#### 3.1.3. Tensile Strength

**Figure 3:**
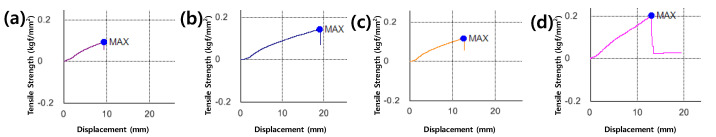
Tensile strength image of (**a**) ref sample, (**b**) Ce sample, (**c**) CeP10 sample and (**d**) CeP10_ISO1 sample.

The strength of hydrogel lenses was determined by measuring the tensile strength of each sample. The results are shown in Figure 3. The tensile strength of ref was 0.094 kgf/mm^2^; that of Ce, Ce10, and CeP10_ISO1 was 0.143, 0.119, and 0.210 kgf/mm^2^, respectively. The tensile strength of the Ce sample that contained cerium(IV) oxide nanoparticles was 52.13% higher than that of the ref sample, and the tensile strength of the TEPI containing sample was 123.40% higher than that of the ref sample. In the same manner as with the refractive index, the tensile strength was significantly higher in the sample that contained TEPI, which suggests that TEPI can be used to enhance the durability of hydrogel lenses. In general, a higher water content is associated with a lower tensile strength. For the CeP10_ISO1 that contained TEPI, the water content and the refractive index were similar to those of the ref sample, whereas the tensile strength was more than two times that of the ref sample. These values demonstrate that adding TEPI and PEGMA to the material of contact lenses will only enhance the material’s tensile strength without affecting its other properties [34].

#### 3.1.4. Tests for Absorbance and Extractables

**Figure 4:**
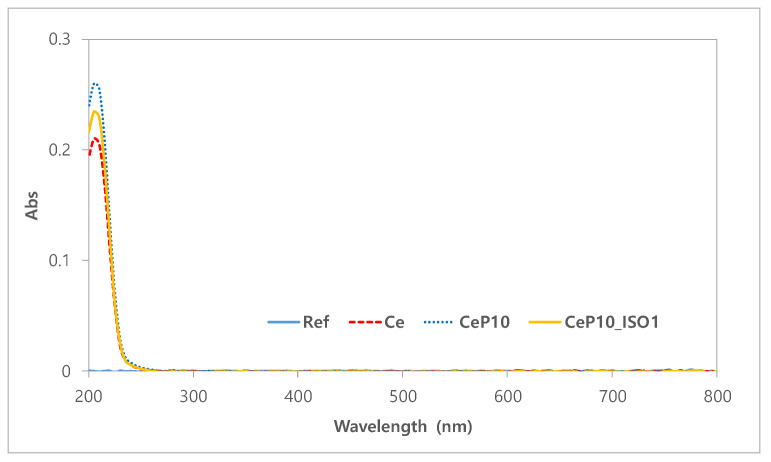
Absorbance values of the hydrated solutions for each sample.

**Figure 5:**
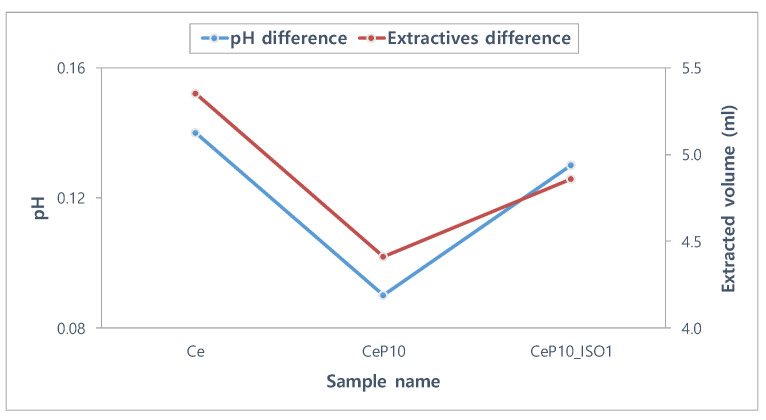
pH and extractables test of samples.

The stability of the lenses is important because the contact lens is a medical device that comes into direct contact with the cornea [35]. Tests for absorbance, pH, and potassium permanganate-reducing substances were conducted to assess the polymerization stability of manufactured hydrogel lenses [36]. Ce, CeP10, and CeP10_ISO1 were compared to evaluate if PEGMA and TEPI used as additives and nanoparticles are not extracted and maintain stable polymerization. The absorbance values of the hydrated solutions for Ce, CeP10, and CeP10_ISO1 were 0.21, 0.26, and 0.23, respectively. The absorbance increased with the addition of PEGMA, but decreased with the addition of TEPI. The absorbance rates of the samples are presented in Figure 4. The pH differences of Ce, CeP10, and CeP10_ISO1 were 0.14, 0.09, and 0.13, respectively, and the presence or absence of a substance causing a potential difference was checked. Additionally, the pH was seen as unaffected by the extractables because its values were below the threshold of 1.5 in all the groups. For the potassium permanganate-reducing substances test, the differences in the control group were 5.35 mL for Ce, 4.41 mL for CeP10, and 4.86 mL for CeP10_ISO1, which suggests that further research is needed to reduce the effect of the extractables. The polymerization status will differ according to the nanoparticles used, and as this will affect the polymerization stability, further research is needed on the polymerization method and condition, as well as on the selection of initiators and cross-linking agents that consider the binding mechanisms. The results of the extractables, by pH and the used reducing substance, are presented in Figure 5.

#### 3.1.5. Antimicrobial Test

**Figure 6:**
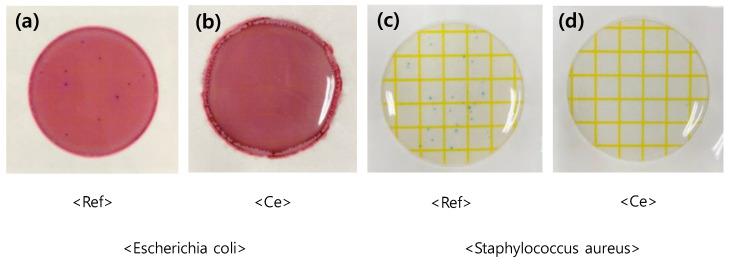
Antimicrobial test of sample of (**a**) ref sample, (**b**) Ce sample, (**c**) Ref sample and (**d**) Ce sample.

To investigate the antibiotic properties of the samples against *Staphylococcus aureus* and *Escherichia coli*, the ref sample, which did not contain cerium(IV) oxide nanoparticles, the basic materials for hydrogel lenses, was set as the control group, and the Ce sample that contained 0.1% cerium(IV) oxide nanoparticles was set as the experimental group. Various previous studies have revealed that cerium nanoparticles have a direct antibacterial effect on cells. In this study, we tried to conduct an experiment on the antibacterial properties of nanoparticles polymerized on contact lenses, and the bacteria that proliferate the most in the eye [37]. The sample to which nanoparticles were added did not show any bacterial growth. The result of the antibiotic test that used dry film media for *S. aureus* and *E. coli* is shown in Figure 6. The result is similar to that of contact lenses that contain silver nanoparticles, which are known to have antibiotic properties [38], and it was demonstrated that cerium oxide nanoparticles can be used as hydrogel hydrophilic lens materials with antibacterial activity.

### 3.2. Surface Property

#### 3.2.1. Wettability

**Figure 7:**
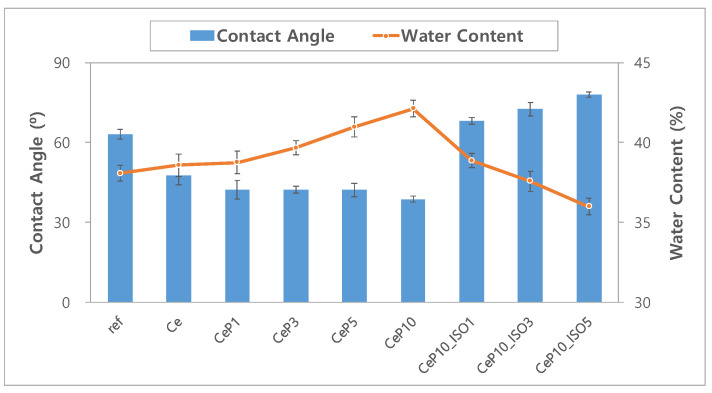
Change in contact angle and water content of samples.

**Figure 8:**
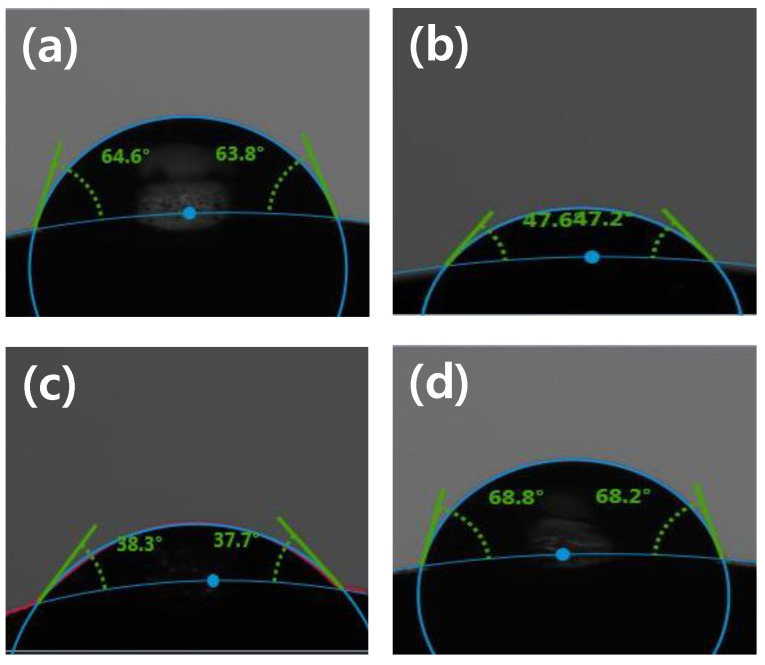
Contact angle image of (**a**) ref sample, (**b**) Ce sample, (**c**) CeP10 sample and (**d**) CeP10_ISO1 sample.

To evaluate the wettability of the samples, contact angles were measured and are shown in Figure 7. The drop images where the contact angle was measured using the Sessile drop method are presented in Figure 8. The contact angles were 63.07° for ref, 47.64° for Ce, and 38.73° for CeP10, which demonstrates that cerium(IV) oxide nanoparticles and PEGMA significantly enhance the wettability of contact lenses. The wettability of a lens is closely related to its comfort, and a wet hydrogel lens tends to cause less dehydration and less tear deposition [39]. The contact angle was bigger in the sample with TEPI than in the sample without TEPI, and was 68.15° in the CeP10_ISO1, similar to that in the conventional ref sample. Generally, a low contact angle indicates high wettability, a high contact angle indicates low wettability, and the 90° contact angle is a criterion for determining hydrophobicity and hydrophilicity. The contact angles of all the samples used in this study were smaller than 90°, which indicates that they are hydrophilic materials. Generally, wettability is proportional to water content, but while the water content of the Ce sample that contained cerium(IV) oxide nanoparticles remained consistent, the wettability was enhanced, which is a very favorable physical property for a person with dry eye syndrome [40,41,42]. Therefore, the use of cerium(IV) oxide nanoparticles and PEGMA enhances the wettability of contact lenses.

#### 3.2.2. SEM and AFM Analyses

**Figure 9:**
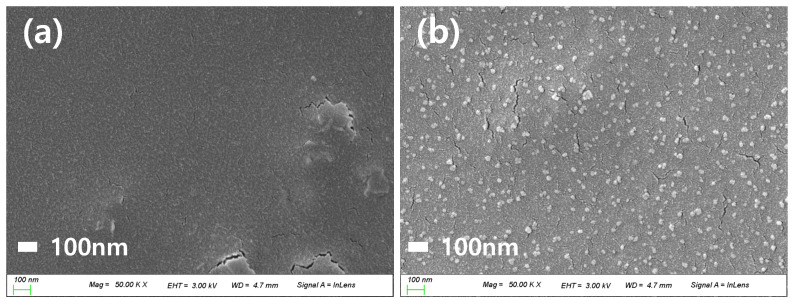
SEM analysis of (**a**) ref sample and (**b**) Ce sample.

**Figure 10:**
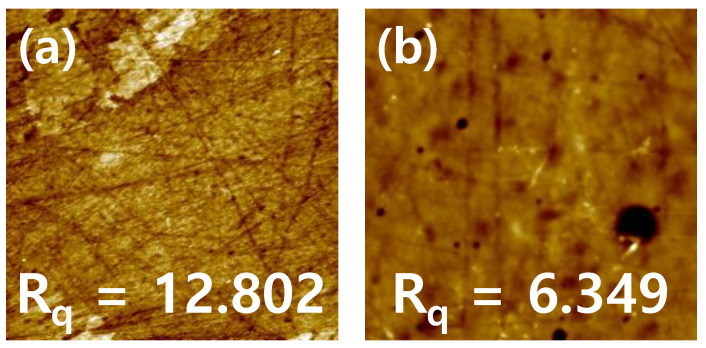
AFM analysis of (**a**) ref sample and (**b**) Ce sample.

The particle dispersion on the lens surface was studied using SEM analysis. The ref sample and CeP10_ISO1 sample lenses, with nanoparticles added to the ref sample, were observed through SEM images, and the size of particles was distributed evenly. The surfaces of the hydrogel lenses were observed through AFM to determine the roughness of the lens surfaces. As a result, the addition of Ce reduced the roughness compared to that of the ref sample. The increase in wettability was attributed to the decrease in surface roughness due to the addition of nanoparticles [43]. The SEM and AFM images of each sample are shown in Figure 9 and Figure 10, respectively.

## 4. Conclusions

This study assessed the physical properties of hydrogel contact lenses when cerium(IV) oxide nanoparticles were added to them, and observed the changes in the physical properties of the lenses according to the concentrations of the PEGMA and TEPI added to their materials. The addition of cerium(IV) oxide nanoparticles to the initial sample did not affect its water content, but enhanced its UV-blocking rate, wettability, and durability. The sample where PEGMA was secondarily added showed enhanced water content, wettability, and visible light transmittance, which are essential hydrogel lens properties. Furthermore, the addition of a small quantity of TEPI slightly increased the contact angle to a level similar to that of the conventional ref, which maintained the physical properties of the hydrophilic hydrogel but increased its tensile strength. Thus, we concluded that the addition of TEPI improved the function of the hydrogel lenses. According to these results, cerium(IV) oxide nanoparticles and TEPI are seen to be diversely applicable as hydrogel contact lens materials that can block UV rays while satisfying the fundamental physical properties of hydrogel ophthalmic lenses.

## Figures and Tables

**Table 1:** Percent composition of samples (unit: wt%).

	HEMA	Ce *	PEGMA	TEPI	EGDMA	AIBN	Total
ref	98.72	-	-	-	0.99	0.2	100
Ce	98.72	0.1	-	-	0.99	0.2	100
CeP1	97.74	0.1	0.98	-	0.99	0.2	100
CeP3	95.84	0.1	2.88	-	0.99	0.2	100
CeP5	94.01	0.1	4.71	-	0.99	0.2	100
CeP10	89.74	0.1	8.98	-	0.99	0.2	100
CeP10_ISO1	88.85	0.1	8.89	0.98	0.99	0.2	100
CeP10_ ISO 3	87.13	0.09	8.72	2.88	0.99	0.2	100
CeP10_ ISO 5	85.47	0.09	8.56	4.71	0.99	0.2	100

* Ce: cerium(IV) oxide nanoparticles.
